# Expansion of Activated Memory CD4^+^ T Cells Affects Infectivity of CCR5-Tropic HIV-1 in Humanized NOD/SCID/JAK3^null^ Mice

**DOI:** 10.1371/journal.pone.0053495

**Published:** 2013-01-02

**Authors:** Kazutaka Terahara, Masayuki Ishige, Shota Ikeno, Yu-ya Mitsuki, Seiji Okada, Kazuo Kobayashi, Yasuko Tsunetsugu-Yokota

**Affiliations:** 1 Department of Immunology, National Institute of Infectious Diseases, Tokyo, Japan; 2 Division of Hematopoiesis, Center for AIDS Research, Kumamoto University, Kumamoto, Japan; 3 Laboratory of Viral Infection II, Kitasato Institute for Life Science, Kitasato University, Tokyo, Japan; University Hospital Zurich, Switzerland

## Abstract

Humanized mice reconstituted with human hematopoietic cells have been developed as an experimental animal model for human immunodeficiency virus type 1 (HIV-1) infection. Myeloablative irradiation is usually performed to augment the engraftment of donor hematopoietic stem cells (HSCs) in recipient mice; however, some mouse strains are susceptible to irradiation, making longitudinal analysis difficult. We previously attempted to construct humanized NOD/SCID/JAK3^null^ (hNOJ) mice, which were not irradiated prior to human HSC transplantation. We found that, over time, many of the reconstituted CD4^+^ T cells expanded with an activated effector memory phenotype. Therefore, the present study used hNOJ mice that were irradiated (hNOJ (IR+)) or not (hNOJ (IR−)) prior to human HSC transplantation to examine whether the development and cellularity of the reconstituted CD4^+^ T cells were influenced by the degree of chimerism, and whether they affected HIV-1 infectivity. Indeed, hNOJ (IR+) mice showed a greater degree of chimerism than hNOJ (IR−) mice. However, the conversion of CD4^+^ T cells to an activated effector memory phenotype, with a high percentage of cells showing Ki-67 expression, occurred in both hNOJ (IR+) and hNOJ (IR−) mice, probably as a result of lymphopenia-induced homeostatic expansion. Furthermore, when hNOJ (IR+) and hNOJ (IR−) mice, which were selected as naïve- and memory CD4^+^ T cell subset-rich groups, respectively, were infected with CCR5-tropic HIV-1 *in vivo*, virus replication (as assessed by the plasma viral load) was delayed; however, the titer subsequently reached a 1-log higher level in memory-rich hNOJ (IR−) mice than in naïve-rich hNOJ (IR+) mice, indicating that virus infectivity in hNOJ mice was affected by the different status of the reconstituted CD4^+^ T cells. Therefore, the hNOJ mouse model should be used selectively, i.e., according to the specific experimental objectives, to gain an appropriate understanding of HIV-1 infection/pathogenesis.

## Introduction

Human immunodeficiency virus type 1 (HIV-1), the causative agent of acquired immunodeficiency syndrome (AIDS) in humans, infects CD4^+^ T cells as well as macrophages and dendritic cells by binding to its primary receptor, CD4, and a co-receptor, usually CCR5 or CXCR4 [1], [2], [3]. Not only is the tropism of HIV-1 determined by its use of either CCR5 or CXCR4, but factors such as cellular activation status, differentiation and maturation status, cell type, and the histological location of the target cells also determine HIV-1 infectivity with respect to its replication, dissemination, and latency [4], [5], [6]. *In vivo* studies are essential if we are to better understand the dynamics of HIV-1 infection and pathogenesis, in addition to improving the trials of putative anti-HIV/AIDS drugs, gene therapy, and vaccines. Therefore, the development of suitable experimental animal models is desirable. Mice reconstituted with human hematopoietic cells, referred to as humanized mice, have recently attracted attention as experimental animal models of HIV-1 infection [7], [8], [9], [10], [11], [12].

At present, bone marrow/liver/thymus (BLT) mice, which are produced by surgical implantation of human fetal thymus and liver tissue into NOD/SCID mice, followed by transplantation of autologous fetal liver CD34^+^ hematopoietic stem cells (HSCs), seem to be an ideal humanized mouse model because they support T cell development in a human thymic environment and generate human MHC-restricted T cell responses *in vivo* [13]. However, due to the ethical issues surrounding the use of fetal organs, studies using BLT mice are limited. Therefore, Rag2^null^IL2Rγ^null^ mice (including BRG (BALB/c-background) and B6RG (C57BL/6-background) mice), or NOD/SCID/IL2Rγ^null^ mice (including NOG (truncated IL-2Rγ chain lacking the intracytoplasmic domain) and NSG (complete absence of IL-2Rγ chain) mice) are conventionally used as recipients of transplanted human HSCs [7], [9], [10], [12], [14], [15], [16]. In addition, BALB/c-Rag1^null^IL2Rγ^null^ mice [17] and NOD/SCID/JAK3^null^ (NOJ) mice [18] have recently been developed as an alternative recipient mouse strain, thereby providing more options for the construction of humanized mice.

Various methods are used to construct humanized mice [12], [14], [19]. The key issue is the efficiency of HSC engraftment [19], [20], [21]. Myeloablative irradiation is conventionally performed to augment the engraftment of donor HSCs within the recipient bone marrow (BM) [22], although irradiation may shorten the life-span of certain strains of mice [17], [18], [22], [23]. Hence, it is difficult to study prolonged HIV-1 infection/pathogenesis using certain strains of irradiated mice. To overcome this problem, Watanabe *et al*. proposed the use of non-irradiated humanized NOG mice, as they have a longer life-span and support HIV-1 infection for over 3 months [22]. For this reason, we attempted to construct a humanized mouse model based on NOJ mice (hNOJ mice) that were not irradiated prior to HSC transplantation. Our preliminary study showed that many of the CD4^+^ T cells that were reconstituted in hNOJ mice expanded with an activated effector memory phenotype over time. Because non-irradiated humanized mice reconstitute human hematopoietic cells less efficiently [22], non-irradiated hNOJ mice may provide a lymphopenic environment that favors lymphopenia-driven homeostatic proliferation (HSP) of T cells. Lymphopenia-induced HSP involves both slowly and rapidly proliferating CD4^+^ T cells: the former remain phenotypically naïve, whereas the latter convert from a naïve to a memory-like phenotype with a greater activation potential [24], [25], [26]. The occurrence of T cell HSP, particularly in the latter case, is supported by other conventional humanized mouse models based on the BRG [27] and NOG [28] strains. Therefore, it is postulated that both the manner and dynamics of HIV-1 infection in humanized mice may be affected by the presence of HSP and, if so, that the humanized mouse model should be used selectively according to the specific experimental objectives.

The aim of the present study was to elucidate whether HSP of CD4^+^ T cells was influenced by the degree of chimerism, and whether it affected HIV-1 infectivity in hNOJ mice. First, we compared the dynamics of reconstituted CD4^+^ T cell cellularity between hNOJ mice that were irradiated (hNOJ (IR+)) or not (hNOJ (IR−)) prior to human HSC transplantation, and characterized them as high and low chimerism groups, respectively. Here, we show that the conversion of CD4^+^ T cells to an activated effector memory phenotype occurred in both hNOJ (IR+) and hNOJ (IR−) mice over time. We also challenged hNOJ (IR+) and hNOJ (IR−) mice, which were selected as naïve- and memory CD4^+^ T cell subset-rich groups, respectively, with CCR5-tropic (R5) HIV-1. The plasma viral load was blunted during the early phase post-challenge, but subsequently reached a 1-log higher level in memory-rich hNOJ (IR−) mice than in naïve-rich hNOJ (IR+) mice. Taken together, the results of the present study provide useful information for evaluating the usefulness of hNOJ mice as a model of HIV-1 infection.

## Methods

### Ethics Statement

Human umbilical cord blood was obtained from the Tokyo Cord Blood Bank (Tokyo, Japan) after receiving written informed consent. Human peripheral blood was obtained from the Blood Bank of Japan Red Cross (Tokyo, Japan) or from healthy adult volunteers after receiving written informed consent. The use of human umbilical cord blood and peripheral blood was approved by the Institutional Ethical Committee of the National Institute of Infectious Diseases (Tokyo, Japan) (Permit Numbers: 127 and 122, respectively). All mice were treated in accordance with the guidelines set down by and approved by the Institutional Animal Care and Use Committee of the National Institute of Infectious Diseases (Permit Numbers: 208022, 109019, 110026, 211033, and 112040).

### Mice

NOD/SCID/JAK3^null^ (NOJ) mice were established as described previously [18] and maintained under specific pathogen-free conditions in the animal facility at the National Institute of Infectious Diseases.

### Construction of Humanized Mice

Human HSCs were isolated from umbilical cord blood using a CD133 MicroBead Kit (Miltenyi Biotec Inc, Auburn, CA). The purity was approximately 90% as assessed by flow cytometric analysis of CD34 expression. Human HSCs (0.5−1×10^5^ cells) were transplanted into the livers of irradiated (1 Gy) or non-irradiated newborn mice within 2 days of birth. The number of hNOJ mice used and the ID number of the donor from which hNOJ mice were derived are listed in Table 1.

**Table 1:** The number of mice used in the present study.

Figure	Number of NOJ mice					Notes
	IR+			IR−		
	Total	Composition (donor #)		Total	Composition (donor #)	
Figure 1						
A	16	2 (D56), 3 (D57), 3 (D59), 1 (D62), 1 (D63), 1 (D65), 1 (D69), 1 (D74), 3 (D80)		28	3 (D47), 2 (D49), 2 (D51), 1 (D52), 2 (D56), 2 (D57), 3 (D59), 2 (D65), 3 (D67), 1 (D68), 2 (D69), 1 (D74), 4 (D80)	
B	7	3 (G65), 1 (G69), 3 (G80)		10	3 (D65), 3 (D69), 4 (D80)	
Figure 2						
A, D, E	22	1 (D56), 3 (D57), 3 (D59), 4 (D62), 5 (D63), 3 (D65), 3 (D80)		13	2 (D56), 2 (D57), 2 (D59), 3 (D65), 4 (D80)	
B, C	5	1 (D54), 2 (D60), 1 (D104), 1 (D105)		6	4 (D54), 1 (D60), 1 (D110)	
Figure 3						
A	1^a^	1 (D65)				^a^Representative of Figure 3B
B	18	3 (D57), 2 (D59), 2 (D62), 5 (D63), 3 (D65), 1 (D69), 2 (D80)		6	3 (D47), 2 (D57), 1 (D80)	
Figure 4						
A	18	^b^		6	^b^	^b^Same as in Figure 3B
B	2	2 (D114)		4	1 (D1), 2 (D3), 1 (D4)	
C	1^c^	1 (D80)				^c^Representative of 4 IR+ mice
D	2	2 (D80)		2	2 (D80)	
E	25	3 (D57), 3 (D59), 4 (D62), 5 (D63), 2 (D64), 3 (D65), 2 (D69), 3 (D80)		21	3 (D47), 2 (D57), 3 (D59), 3 (D65), 3 (D67), 1 (D68), 3 (D69), 3 (D80)	
Figure 5						
A	12	3 (D65), 1 (D90), 6 (D101), 1 (D113), 1 (D114)		8	2 (D59), 1 (D65), 1 (D74), 1 (D85), 3 (D92)	
B	6	2 (D80), 1 (D101), 2 (D113), 1 (D114)		6	3 (D80), 3 (D92)	
C	1^d^	1 (G113)				^d^Representative of Figure 5D
D	3	2 (D113), 1 (D114)		3	3 (D92)	
Figure 6						
A, B	18	^b^		6	2 (D57), 1 (D59), 2 (D69), 1 (D80)	^b^Same as in Figure 3B
C	1^e^	1 (D65)				^e^Representative of Figure 6D
D, E	3	3 (D65)		1	1 (D59)	
Figure 7						
A, B, C	7	7 (D101)		8	2 (D13), 1 (D23), 5 (D122)	
D	6^f^	6 (D101)		7^f^	2 (D13), 1 (D23), 4 (D122)	^f^Included in Figure 7A, B, C
Figure 8				3	3 (D33)	

### Cell Preparation

Human peripheral blood mononuclear cells (PBMCs) were separated by a Ficoll-Hypaque density gradient (Lymphosepal; IBL, Gunma, Japan). For hNOJ mice, peripheral blood, spleens and BM were collected and the red blood cells were lysed in ACK buffer (0.15 M NH_4_Cl, 1 mM KHCO_3_, and 0.1 mM EDTA-2Na; pH 7.2−7.4). In some cases, CD4^+^ T cells from human PBMCs or hNOJ splenocytes were negatively selected using an EasySep Human CD4^+^ T cell Enrichment Kit (StemCell Technologies, Vancouver, BC, Canada), or a combination of this kit and a StemSep Mouse/Human Chimera Enrichment Kit (StemCell Technologies), respectively. The purity was ≥95% as assessed by flow cytometry.

### Flow Cytometry

Cells were stained with fluorescence-conjugated monoclonal antibodies as described previously [29]. The following antibodies were used for flow cytometry in various combinations: FITC-conjugated anti-mouse CD45 (30-F11), anti-human CD34 (581), and CD195/CCR5 (HEK/1/85a); PE-conjugated anti-human CD19 (HIB19), CD150 (A12(7D4)), CD184/CXCR4 (12G5) and IFN-γ (4S.B3); PerCP-conjugated anti-human CD3 (UCHT1), CD4 (RPA-T4), CD8a (RPA-T8), and HLA-DR (L243); PE-Cy7-conjugated anti-human CD3 (UCHT1); APC-conjugated anti-human CD8a (RPA-T8) and CD45RA (HI100); Alexa Fluor 647-conjugated anti-human CD25 (BC96); Alexa Fluor 700-conjugated anti-human CD4 (OKT4), CD27 (O323) and CD69 (FN50); Pacific Blue-conjugated anti-human CD3 (UCHT1), CD4 (RPA-T4), and CD45 (HI30) (all purchased from BioLegend, San Diego, CA). FITC-conjugated anti-human Ki-67 (B56) and PE-Cy7-conjugated anti-human CD197/CCR7 (3D12) were purchased from BD Biosciences (San Diego, CA). APC-conjugated anti-human CD14 (TÜK4) was purchased from Miltenyi Biotec Inc. Anti-human CD11a (TS1/22.1.1.13) and CD38 (OKT10) antibodies were prepared from hybridoma cells (ATCC Nos. HB202 and CRL8022, respectively) and were conjugated with Alexa Fluor 647 and Alexa Fluor 700, respectively, using Alexa Fluor succinimidyl esters (Invitrogen, Carlsbad, CA). Dead cells were stained with propidium iodide or with a LIVE/DEAD Fixable Dead Cell Stain Kit (L34957; Invitrogen) and were gated out during analysis. Intracellular staining for Ki-67 and IFN-γ was performed using a BD Cytofix/Cytoperm Fixation/Permeabilization Solution (BD Biosciences) or a FIX & PERM Fixation and Permeabilization Kit (Invitrogen). Absolute cell counts in the peripheral blood of hNOJ mice were determined using Trucount tubes (BD Biosciences). Data were collected using a FACSCanto II (BD Biosciences) and analyzed using FACSDiva software (BD Biosciences) or FlowJo software (Tree Star, San Carlos, CA).

### *Ex vivo* IFN-γ Production in CD4^+^ T Cells Induced by PMA/ionomycin Stimulation

Purified CD4^+^ T cells were stimulated *ex vivo* with or without 20 ng/ml phorbol 12-myristate 13-acetate (PMA; Sigma-Aldrich, St. Louis, Mo) and 1 µg/ml ionomycin (Sigma-Aldrich) in RPMI medium containing 10% heat-inactivated fetal bovine serum, 100 µg/ml penicillin, 100 µg/ml streptomycin, 2 mM L-glutamine, 5 µg/ml brefeldin A, and 2 µM monensin at 37°C for 4 h. Intracellular IFN-γ was analyzed by flow cytometry as described above. Because PMA treatment downmodulates CD4 expression [30], and to distinguish CD4^+^ T cells from CD8^+^ T cells (a minor contaminant in the purified CD4^+^ T cell fraction), CD3^+^CD8^−^ T cells were denoted as CD4^+^ T cells in this experiment.

### Detection of Cytokines in the Plasma

IL-2, IL-7, and IL-15 levels in the plasma from routinely collected peripheral blood samples were measured using a Milliplex MAP Human Cytokine/Chemokine Panel (Merck Millipore Japan, Tokyo, Japan) on a MAGPIX platform (Merck Millipore Japan).

### Fusion Assay

A fusion assay was performed using HIV-1 possessing β-lactamase-Vpr chimeric proteins (BlaM-Vpr) and CD4^+^ T cells loaded with CCF2 dye, a fluorescent substrate for β-lactamase, as previously described [31], [32]. In brief, R5 HIV-1_NL-AD8-D_ [29] containing BlaM-Vpr (HIV-1_NL-AD8-D-BlaM-Vpr_) was obtained by cotransfecting 293T cells with pNL-AD8-D plus pMM310, encoding *Escherichia coli* β-lactamase fused to the amino terminus of Vpr [33]. The purified CD4^+^ T cells (1×10^6^ cells) were infected with 200 ng of p24-measured amounts of HIV-1_NL-AD8-D-BlaM-Vpr_ by spinoculation at 1200×*g* for 2 h at 25°C as previously described [34]. After spinoculation, cells were washed and then incubated in RPMI containing 10% heat-inactivated fetal bovine serum for 2 h at 37°C to induce viral fusion. After fusion, cells were washed and loaded with CCF2-AM for 1 h at RT using a GeneBLAzer *In Vivo* Detection Kit (Invitrogen). The dye-loaded cells were incubated overnight at RT and subjected to flow cytometry. Cells permissive for HIV-1 fusion were detected at a fluorescence emission spectrum of 447 nm after excitation with a 405-nm violet laser in a FACSCanto II.

### *In vivo* HIV-1 Infection of hNOJ Mice

hNOJ mice were challenged intravenously with 200 ng of p24-measured amounts of R5 HIV-1_NL-AD8-D_, which express DsRed [29]. Peripheral blood was harvested from the HIV-1-challenged hNOJ mice on a weekly basis. All animal experiments with highly pathogenic viruses were conducted in a biosafety level 3 containment facility.

### Detection of Plasma Viral RNA by Quantitative Real-time RT-PCR

Viral RNA was extracted from the plasma and purified using a QIAamp Viral RNA Mini Kit (Qiagen, Valencia, CA). The RNA was subjected to quantitative real-time RT-PCR using a SuperScript III Platinum One-Step Quantitative RT-PCR System (Invitrogen) with the following set of HIV-1 gag primers and probe [35]: forward primer, HIVgag638 (+) (5′-CTCTCGACGCAGGACTCGGCTTGCT-3′); reverse primer, HIVgag803 (−) (5′-GCTCTCGCACCCATCTCTCTCCTTCTAGCC-3′); and HIV-1 gag probe, TaqMan 720R748 (FAM-5′-GCAAGAGGCGAGRGGCGGCGACTGGTGAG-3′-BHQ-1). PCR was performed using an Mx3000P PCR system (Stratagene, La Jolla, CA). The detection limit was set at 5000 copies/ml plasma using samples obtained from HIV-1_NL-AD8-D_-challenged NOJ mice that were not transplanted with HSCs.

### Statistical Analysis

The significance of the data was evaluated using an unpaired *t* test, a paired *t* test, the Mann-Whitney U test, the Wilcoxon signed rank test, Spearman’s rank correlation coefficient, or Tukey’s or Bonferroni multiple comparison tests based on the normality and variance of the data compared, or the Log-rank test (see individual Figure Legends). Prism ver.4 software (GraphPad Software, Inc., San Diego, CA) was used for all analyses. *P*<0.05 was considered statistically significant.

## Results

### Influence of Irradiation on the Survival and Growth of hNOJ Mice

**Figure 1:**
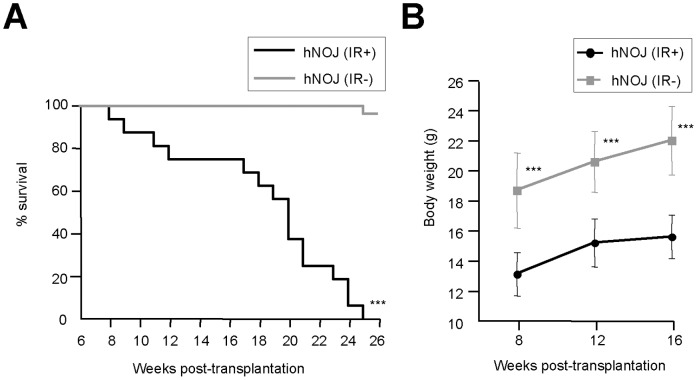
Influence of irradiation on the survival and growth of hNOJ mice. Newborn NOJ mice (1−2 days after birth) were irradiated (1 Gy) or not before transplantation with CD34^+^CD133^+^ HSCs isolated from human cord blood. (A) Survival curves for hNOJ (IR+) and hNOJ (IR−) mice (*n* = 16 and *n* = 28, respectively). Significant differences (^***^ *P*<0.001) were determined by the log-rank test. (B) Changes in the body weight of hNOJ (IR+) and hNOJ (IR−) mice (*n* = 7 and *n* = 10, respectively). Data are expressed as the mean ± SD. Significant differences (^***^ *P*<0.001) were determined by the unpaired *t* test.

We initially examined how the irradiation of recipient mice influences their survival and growth. Because infant mice were sometimes cannibalized and abandoned by their mothers, and the death of an infant could not always be attributed to irradiation, we started monitoring weaned mice from 6 wk post-transplantation. There was a significant difference between the survival curves of irradiated hNOJ (hNOJ (IR+)) and non-irradiated hNOJ (hNOJ (IR−)) mice (*n* = 16 and *n* = 28, respectively, *P*<0.001) (Figure 1A). At 16 wk post-transplantation, the survival rate of hNOJ (IR+) mice dramatically declined (median survival: 20.0 wk) and none survived beyond 25 wk post-transplantation (Figure 1A). However, obvious signs and symptoms of illness, such as wasting, weakness, diarrhea, hunchback posture, and alopecia, were not observed during their lifetime; although the growth of hNOJ (IR+) mice (*n* = 7) was significantly stunted compared with that of hNOJ (IR−) mice (*n* = 10) (Figure 1B). Although the life-span of the hNOJ (IR+) mice used in the present study was shorter than that reported previously [18], probably because of the environmental conditions in our animal facility, these results demonstrated that irradiation apparently induces high mortality and low growth in hNOJ mice. Therefore, in the present study, the averaged data obtained from hNOJ (IR+) mice surviving up until 16 wk post-transplantation are reported for all the following experiments.

### Development of Human Hematopoietic Cells in hNOJ Mice

**Figure 2:**
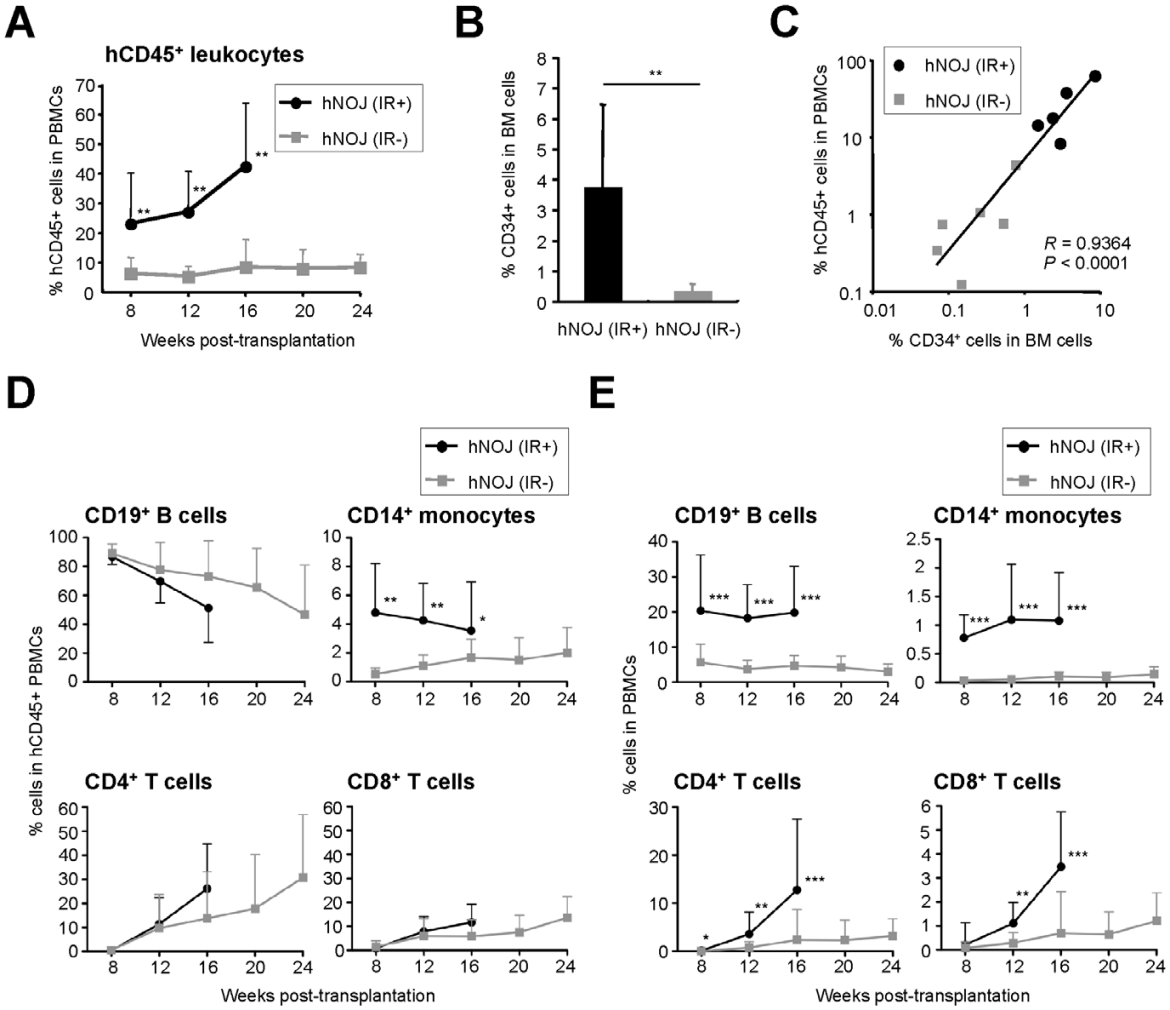
Development of human hematopoietic cells in hNOJ mice. (A) Changes in the percentage of human CD45^+^ (hCD45^+^) cells within the PBMC population from hNOJ (IR+) and hNOJ (IR−) mice (*n* = 22 and *n* = 13, respectively). Data are expressed as the mean ± SD. Significant differences (^**^ *P*<0.01) were determined by the Mann-Whitney U test. (B) Percentage of human CD34^+^ cells within the BM cells isolated from hNOJ (IR+) and hNOJ (IR−) mice (*n* = 5 and 6, respectively) at 8 wk post-transplantation. Data are expressed as the mean ± SD. Significant differences (^**^ *P*<0.01) were determined by the Mann-Whitney U test. (C) Association between the percentage of hCD45^+^ cells within the PBMC population and that of CD34^+^ cells within the BM cells of hNOJ (IR+) and hNOJ (IR−) mice at 8 wk post-transplantation (11 plots from five hNOJ (IR+) and six hNOJ (IR−) mice). Spearman’s rank correlation coefficient was used for statistical analysis. (D, E) Changes in the percentage of human CD19^+^ B cells, CD14^+^ monocytes, CD4^+^ T cells (CD3^+^CD4^+^CD8^−^ cells), and CD8^+^ T cells (CD3^+^CD4^−^CD8^+^ cells) within the peripheral blood hCD45^+^ cell population (D) or total PBMC populstion (E) from hNOJ (IR+) and hNOJ (IR−) mice (*n* = 22 and *n* = 13, respectively). Data are expressed as the mean ± SD. Significant differences (^*^ *P*<0.05, ^**^ *P*<0.01, ^***^ *P*<0.001) were determined by the Mann-Whitney U test.

Reconstitution of human hematopoietic cells (i.e., chimerism) in hNOJ mice was investigated by flow cytometry using peripheral blood samples collected routinely (every 4 wk) after 8 wk post-transplantation. hNOJ (IR+) mice (*n* = 22) showed higher chimerism (according to the percentage of human CD45^+^ (hCD45^+^) leukocytes within the total PBMC population) than hNOJ (IR−) mice (*n* = 13) during the course of the experiment (Figure 2A).

Furthermore, we examined the effects of irradiation on the engraftment of HSCs in the BM of hNOJ mice in which hCD45^+^ leucocytes were observed. hNOJ (IR+) mice showed a significantly higher percentage of CD34^+^ cells within the total BM cell population (an average of 3.7%; *n* = 5) than hNOJ (IR−) mice (an average of 0.3%; *n* = 6) at 8 wk post-transplantation (Figure 2B). The percentage of CD34^+^ cells within the total BM cell population was positively correlated with the percentage of hCD45^+^ leukocytes within the total PBMC population at 8 wk post-transplantation (*R* = 0.9364, *P*<0.001, *n* = 11 [five hNOJ (IR+) and six hNOJ (IR−) mice]; Figure 2C). These results indicate that irradiation augments the chimerism of human hematopoietic cells due to the enhanced engraftment of HSCs in the BM of hNOJ mice.

Next, we investigated the development of hematopoietic cell subpopulations in hNOJ (IR+) and hNOJ (IR−) mice (*n* = 22 and *n* = 13, respectively). The reconstituted hCD45^+^ leukocytes within the total PBMC populations from hNOJ (IR+) and hNOJ (IR−) mice comprised CD19^+^ B cells, CD14^+^ monocytes, and CD3^+^ T cells (including CD4^+^ and CD8^+^ T cells); however, the development of each subpopulation was different (Figure 2D). CD19^+^ B cells developed early in both hNOJ (IR+) and hNOJ (IR−) mice, as observed at 8 wk post-transplantation, before gradually declining over time (Figure 2D, left upper panel). CD14^+^ monocytes also developed during the early phase post-transplantation in hNOJ (IR+) mice, but were rare in hNOJ (IR−) mice. In general, the percentage of all the subpopulations was significantly lower in hNOJ (IR−) mice than in hNOJ (IR+) mice up until 16 wk post-transplantation, although the percentage of CD14^+^ monocytes in hNOJ (IR−) mice gradually increased with time (Fig. 2D, right upper panel). In apparent contrast to the earlier development of CD19^+^ B cells, the development of CD3^+^ T cells was delayed; they were clearly apparent at around 12 wk post-transplantation in both hNOJ (IR+) and hNOJ (IR−) mice (Figure 2D, left and right lower panels), consistent with previous reports of other conventional humanized mouse models [16], [20], [36], [37]. The percentage of CD3^+^ T cells reflected that of CD4^+^ T cells; the level of CD4^+^ T cells was higher than that of CD8^+^ T cells, and their developmental level tended to be higher in hNOJ (IR+) mice than in hNOJ (IR−) mice, although no statistically significant differences were observed between hNOJ (IR+) and hNOJ (IR−) mice (Figure 2D, lower left and right panels). However, both the CD4^+^ and CD8^+^ T cell percentages within the total PBMC population in hNOJ (IR+) mice were significantly higher than those in hNOJ (IR−) mice at almost time-points up until 16 wk post-transplantation (Figure 2E, lower left and right panels). It is noteworthy that reconstitution of CD4^+^ T cells was detected in more hNOJ (IR+) mice (18 of 22 mice) than hNOJ (IR−) mice (4 of 13 mice) at 8 wk post-transplantation, although the averaged percentages were slightly lower (0.12±0.33% and 0.02±0.03% CD4^+^ T cells, respectively, within the total PBMC population). The percentages of other human hematopoietic cells within the total PBMC population, such as CD19^+^ B cells, CD14^+^ monocytes and CD8^+^ T cells, were also higher in hNOJ (IR+) mice than in hNOJ (IR−) mice at all time-points up to 16 wk post-transplantation (Figure 2E). Taken together, these results indicate that irradiation contributes to the greater reconstitution of human hematopoietic cells in these humanized mice.

### Differentiation of CD4^+^ T Cells in hNOJ Mice

A reliable differentiation pathway for human CD4^+^ T cells has been proposed: an antigen-experienced naïve subset differentiates into a central memory (CM) subset, followed by differentiation into an effector memory (EM) subset, before finally differentiating into a terminal effector subset [38], [39]. To investigate the differentiation stage of CD4^+^ T cells reconstituted in hNOJ mice, we used hNOJ (IR+) and hNOJ (IR−) mice in which CD4^+^ T cells had developed by 12 wk post-transplantation.

**Figure 3:**
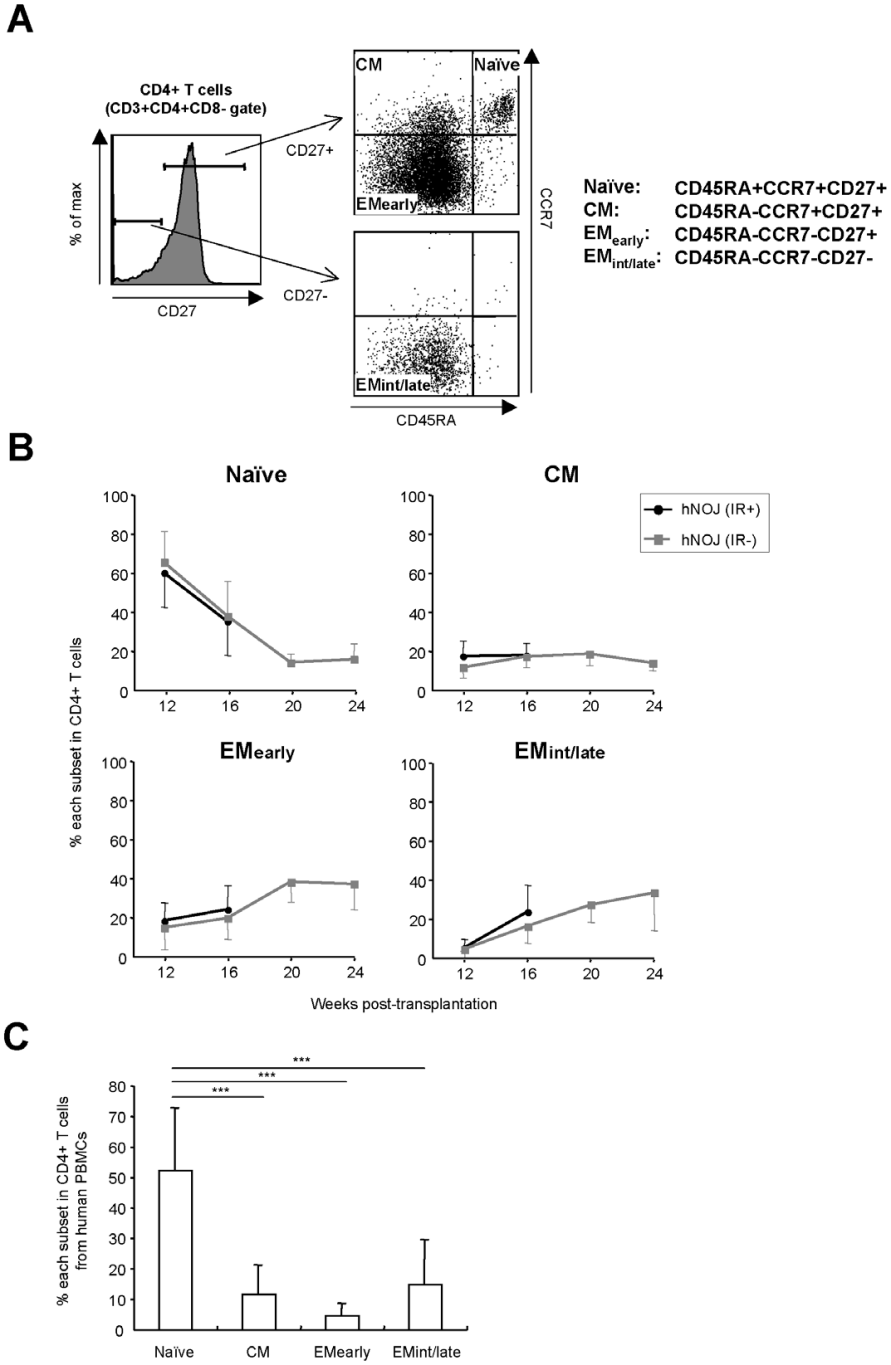
Differentiation of CD4^+^ T Cells in hNOJ mice. (A) Naïve, CM, EM_early_, and EM_int/late_ subsets of CD4^+^ T cells (gated on CD3^+^CD4^+^CD8^−^) were defined as CD45RA^+^CCR7^+^CD27^+^, CD45RA^−^CCR7^+^CD27^+^, CD45RA^−^CCR7^−^CD27^+^, and CD45RA^−^CCR7^−^CD27^−^, respectively, by flow cytometry. (B) Changes in the percentage of naïve, CM, EM_early_, and EM_int/late_ subsets within the peripheral blood CD4^+^ T cell populations isolated from hNOJ (IR+) and hNOJ (IR−) mice (*n* = 18 and *n* = 6, respectively). Data are expressed as the mean ± SD. (C) Percentage of CM, EM_early_, and EM_int/late_ subsets within human peripheral blood CD4^+^ T cells. Data are expressed as the mean ± SD (*n* = 10). Significant differences (^***^ *P*<0.001) were determined by Tukey’s multiple comparison test.

As shown in Figure 3A, we divided CD4^+^ T cells into four subsets based on their expression of cell surface antigens as previously reported: naïve (CD45RA^+^CCR7^+^CD27^+^), CM (CD45RA^−^CCR7^+^CD27^+^), EM_early_ (CD45RA^−^CCR7^−^CD27^+^) and EM_int/late_ (CD45RA^−^CCR7^−^CD27^−^) [38], [40], [41]. Flow cytometric analysis using PBMCs routinely collected from hNOJ (IR+) and hNOJ (IR−) mice (*n* = 18 and *n* = 6, respectively) demonstrated that the percentage of each CD4^+^ T cell subset showed similar changes in hNOJ (IR+) and hNOJ (IR−) mice by 16 wk post-transplantation (Figure 3B). The naïve subset was dominant at 12 wk post-transplantation; however, the percentage decreased markedly, reaching a steady level of approximately 20% at 20 wk post-transplantation in hNOJ (IR−) mice. In sharp contrast with the changes over time observed for the naïve subset, the percentages of the EM_early_ and EM_int/late_ subsets in hNOJ (IR−) mice gradually increased over time. The percentage of the CM subset in hNOJ (IR−) mice remained unchanged at 10−20% throughout the course of the experiment. The percentage of each subset in the hNOJ mice during the early phase (12 wk) post-transplantation was almost identical to that in human PBMCs, in which the naïve subset was also dominant (approximately 50%, *n* = 10) (Figure 3C).

### Activation Status of CD4^+^ T Cells in hNOJ Mice

**Figure 4:**
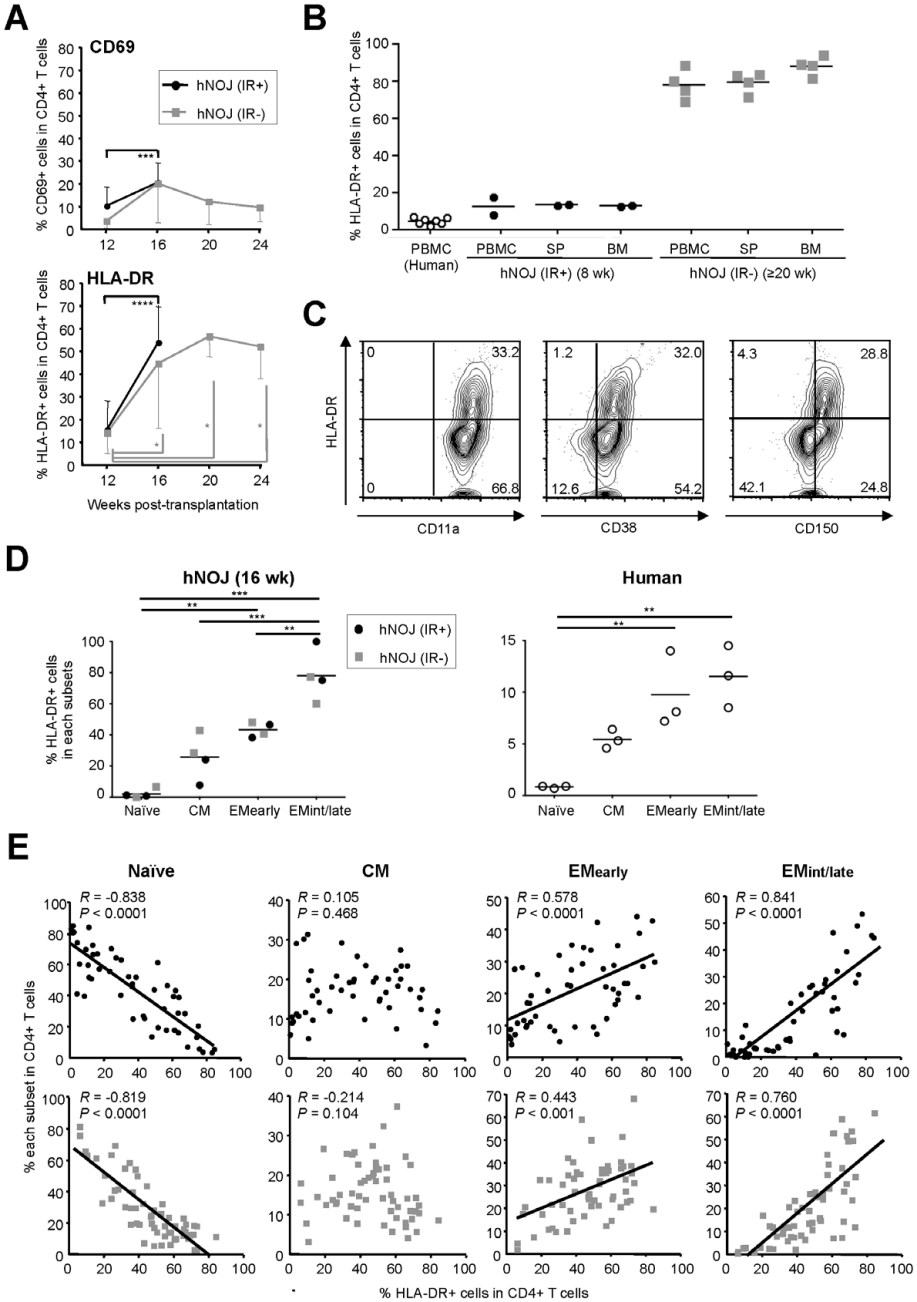
Activation status of CD4^+^ T Cells in hNOJ mice. (A) Changes in the percentage of CD69^+^ (upper) and HLA-DR^+^ (lower) cells within the peripheral blood CD4^+^ T cells isolated from hNOJ (IR+) and hNOJ (IR−) mice (*n* = 18 and *n* = 6, respectively). Data are expressed as the mean ± SD. Significant differences (^*^ *P*<0.05, ^****^ *P*<0.0001) were determined using the Wilcoxon signed rank test or a paired *t* test. (B) Percentage of HLA-DR^+^ cells within the CD4^+^ T cell populations isolated from PBMCs, spleens (SP), and BM from hNOJ (IR+) mice at 8 wk post-transplantation (*n* = 2) and hNOJ (IR−) mice at ≥20 wk post-transplantation (*n* = 4), and from human PBMCs (*n* = 7). Data are plotted individually. The black line represents the mean. (C) Expression of HLA-DR and other activation markers (CD11a, CD38, and CD150) by peripheral blood CD4^+^ T cells. Representative flow cytometry profiles are shown from one of four hNOJ (IR+) mice at 16 wk post-transplantation. (D) Percentage of HLA-DR^+^ cells in the naïve, CM, EM_early_, and EM_int/late_ subsets of peripheral blood CD4^+^ T cells isolated from hNOJ mice at 16 wk post-transplantation (*n* = 4; two hNOJ (IR+) and two hNOJ (IR−) mice) and from humans (*n* = 3). Data are expressed as the mean ± SD. Significant differences (^**^ *P*<0.01, ^***^ *P*<0.001) were determined by Tukey’s multiple comparison test. (E) Association between the percentage of each CD4^+^ T cell subset and that of HLA-DR^+^ cells in the peripheral blood CD4^+^ T cell population. Data were obtained from peripheral blood samples routinely collected from hNOJ (IR+) and hNOJ (IR−) mice within 28 wk post-transplantation (50 data points obtained from 25 mice (upper panels) and 59 data points obtained from 21 mice (lower panels), respectively). Spearman’s rank correlation coefficient was used for statistical analysis.

The activation status of the reconstituted CD4^+^ T cells was assessed by flow cytometric analysis of PBMCs isolated from hNOJ (IR+) and hNOJ (IR−) mice (*n* = 18 and *n* = 6, respectively), based on their expression of early and late activation markers (CD69 and HLA-DR, respectively). We found that the percentage of CD69^+^CD4^+^ T cells increased in hNOJ (IR+) mice at 16 wk post-transplantation compared with that observed at 12 wk post-transplantation (*P* = 0.0007). In hNOJ (IR−) mice, the percentage of CD69^+^CD4^+^ T cells appeared to show a transient increase at 16 wk post-transplantation; however, no significant differences were observed between 16 wk post-transplantation and the other time-points (Figure 4A, upper panel). By contrast, the percentage of HLA-DR^+^CD4^+^ T cells increased in both hNOJ (IR+) and hNOJ (IR−) mice at 16 wk post-transplantation compared with 12 wk post-transplantation, and the difference was statistically significant (*P*<0.0001 and *P*<0.0313). Also, this increase was maintained at about 50−60% beyond 16 wk post-transplantation in hNOJ (IR−) mice (Figure 4A, lower panel). To assess the activation status of CD4^+^ T cells in other organs, such as the spleen and BM, we next performed an experiment using hNOJ (IR+) mice at 8 wk post-transplantation and hNOJ (IR−) mice at ≥20 wk post-transplantation as representative examples of early- and late-phase, respectively, CD4^+^ T cell development. We found that the expression of HLA-DR by peripheral blood CD4^+^ T cells isolated from hNOJ mice was consistent with that by cells isolated from the spleen and BM: lower expression of HLA-DR (7.7% and 17.1% in two hNOJ (IR+) mice) during the early phase (8 wk) post-transplantation, which is also equivalent to that in human PBMCs, and higher expression (68.7−93.8% in four hNOJ (IR−) mice) during the later phase (≥20 wk) post-transplantation (Figure 4B). To address whether HLA-DR expression actually reflects the activation status of reconstituted CD4^+^ T cells, we examined the expression of other activation markers: CD11a, CD38, and CD150. Figure 4C shows representative flow cytometry profiles obtained using peripheral blood CD4^+^ T cells isolated from one of four hNOJ (IR+) mice at 16 wk post-transplantation. HLA-DR^+^CD4^+^ T cells also expressed these markers, confirming the highly activated status of the HLA-DR^+^CD4^+^ T cells reconstituted in hNOJ mice.

We further investigated whether the activation status of CD4^+^ T cells was associated with their differentiation stage. When the total percentage of HLA-DR^+^ cells within the peripheral blood CD4^+^ T cell subset populations was examined, we found that the memory CD4^+^ T cell subsets, particularly the EM_int/late_ subset, made up a significantly higher proportion of this percentage than the naïve subset (*n* = 4; two hNOJ (IR+) and two hNOJ (IR−) mice; Figure 4D). The different HLA-DR expression patterns between the CD4^+^ T cell subsets were similar in human PBMCs (*n* = 3), although the expression levels were much lower than those in hNOJ mice (Figure 4D). To further address this differentiation stage-dependent level of HLA-DR expression by CD4^+^ T cells, we compared the percentage of each CD4^+^ T cell subset with the percentage of HLA-DR^+^ cells within the total CD4^+^ T cell population using data obtained from peripheral blood samples routinely collected from hNOJ (IR+) and hNOJ (IR−) mice (*n* = 25 and *n* = 21, respectively) within 25 wk and 28 wk post-transplantation, respectively. We found that the percentage of HLA-DR^+^ cells positively correlated with that of the EM_early_ and EM_int/late_ subsets, and inversely correlated with that of the naïve subset, in both hNOJ (IR+) and hNOJ (IR−) mice (Figure 4E). However, there was no significant correlation between the percentage of HLA-DR^+^ cells and that of the CM subset in either hNOJ (IR+) or hNOJ (IR−) mice, indicating an intermediate stage at which cells are acquiring the activation phenotype (Figure 4E). Taken together, reconstituted CD4^+^ T cells in hNOJ mice express a unique phenotype compared with human peripheral blood CD4^+^ T cells: activated EM subsets predominate with time after transplantation.

### Homeostatic Peripheral Expansion of CD4^+^ T Cells May Occur in hNOJ Mice

**Figure 5:**
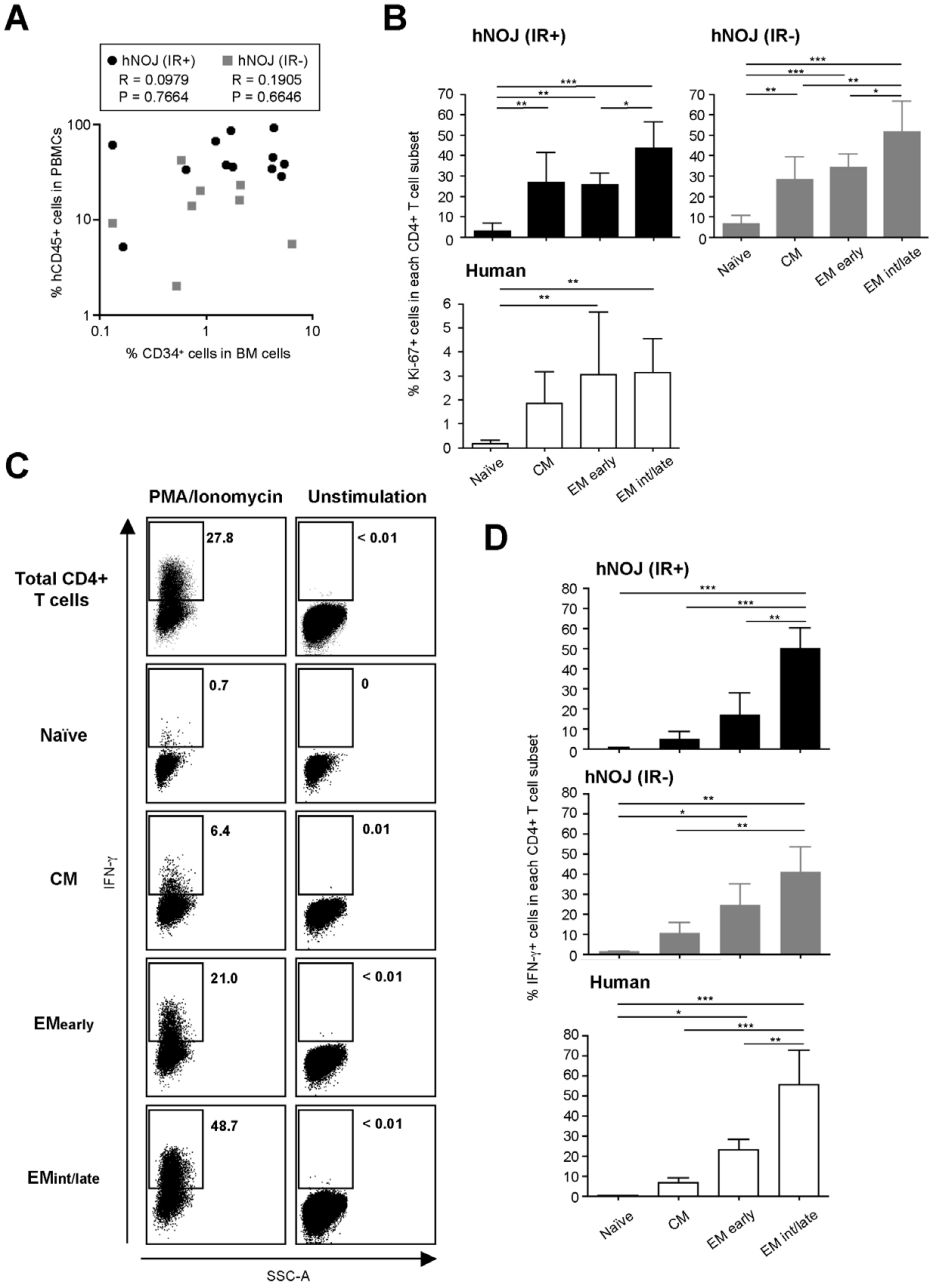
Possible occurrence of HSP of CD4^+^ T Cells in hNOJ mice. (A) Association between the percentage of hCD45^+^ cells within the PBMC population and that of CD34^+^ cells in the BM cells from hNOJ (IR+) and hNOJ (IR−) mice at ≥16 wk post-transplantation (*n* = 12 and *n* = 8, respectively). Spearman’s rank correlation coefficient was used for statistical analysis. (B) Percentage of Ki-67^+^ cells among naïve, CM, EM_early_, and EM_int/late_ subsets of splenic CD4^+^ T cells from hNOJ (IR+) and hNOJ (IR−) mice at ≥16 wk post-transplantation (*n* = 6 and *n* = 6, respectively) and from human PBMCs (*n* = 10). Data are expressed as the mean ± SD. Significant differences (^*^ *P*<0.05, ^**^ *P*<0.01, ^***^ *P*<0.001) were determined by Tukey’s multiple comparison test. (C and D) *Ex vivo* IFN-γ production by CD4^+^ T cells after stimulation with PMA/ionomycin. CD4^+^ T cells were prepared from the spleens of hNOJ (IR+) and hNOJ (IR−) mice at ≥16 wk post-transplantation or from human PBMCs. (C) Representative flow cytometry profiles showing the proportion of IFN-γ^+^ cells within each of the CD4^+^ T cell subsets from a hNOJ (IR+) mouse at 16 wk post-transplantation. (D) Cumulative data showing the percentage of IFN-γ^+^ cells within each of the CD4^+^ T cell subsets from hNOJ (IR+) and hNOJ (IR−) mice and humans (*n* = 3, *n* = 3, and *n* = 4, respectively). Data are expressed as the mean ± SD. Significant differences (^*^ *P*<0.05, ^**^ *P*<0.01, ^***^ *P*<0.001) were determined by Tukey’s multiple comparison test.

When the percentage of hCD45^+^ leukocytes within the total PBMC population was compared with that of CD34^+^ cells within the total BM cell population during the later phase (≥16 wk) post-transplantation, no significant correlation was observed in either hNOJ (IR+) or hNOJ (IR−) mice (*n* = 12 and *n* = 8, respectively) (Figure 5A). These results prompted us to assume that CD4^+^ T cell proliferation/expansion was predominant in hNOJ mice during the late phase post-transplantation. Therefore, we next examined the expression of a proliferation marker, Ki-67, by reconstituted CD4^+^ T cells using flow cytometry to assess the proliferative capacity at different differentiation stages. CD4^+^ T cells were prepared from the spleens of hNOJ (IR+) and hNOJ (IR−) mice during the later phase (≥16 wk) post-transplantation (*n* = 6 and *n* = 6, respectively). CD4^+^ T cells isolated from human PBMCs (*n* = 10) were used as a control. The results showed that Ki-67^+^ cells were present within the memory CD4^+^ T cell subsets (CM, EM_early_ and EM_int/late_) at higher percentages than within the naïve subset (Figure 5B, upper two panels). A higher percentage of Ki-67^+^ cells was also observed in the memory subsets within the human PBMC population than in the naïve subset; however, the levels were much lower than those observed in the hNOJ mice (Figure 5B, lower panel). These results support the notion that increased CD4^+^ T cell proliferation/expansion occurs in hNOJ mice during the later phase post-transplantation.

HSP of CD4^+^ T cells involves both slow and rapid proliferation pathways. The slow pathway is IL-7-dependent and results in limited cell activation and differentiation, whereas the latter is T cell receptor (TCR)-dependent and IL-7-independent and results in robust cell activation and differentiation into a memory phenotype with the capacity to produce IFN-γ [25], [26]. The term “HSP” is generally used to indicate the former; the latter is often referred to as “spontaneous HSP” [25] or “homeostatic peripheral expansion (HPE)” [26]. It was assumed that HPE-type HSP might occur in hNOJ mice, as described elsewhere [27], [28]; therefore, we examined the IFN-γ-producing capacity of CD4^+^ T cells isolated from the spleens of hNOJ (IR+) and hNOJ (IR−) mice (*n* = 3 and *n* = 3, respectively) during the later phase (16 and 26 wk, respectively) post-transplantation by stimulating them with or without PMA plus ionomycin. Flow cytometric analysis of intracellular IFN-γ staining showed substantial numbers of IFN-γ-producing cells within the EM_int/late_ subset after PMA/ionomycin stimulation; the data were similar when CD4^+^ T cells from human PBMCs were used in the experiment (*n* = 4) (Figure 5C and 5D). No IFN-γ-producing cells were detectable in any of the CD4^+^ T cell subsets from hNOJ (IR+) and hNOJ (IR−) mice, or in human PBMCs without PMA/ionomycin stimulation (Figure 5C and data not shown).

Next, we measured the concentrations of cytokines (IL-2, IL-7, and IL-15, all of which are involved in CD4^+^ T cell proliferation [24], [42]), in plasma prepared from peripheral blood samples taken from hNOJ (IR+) and hNOJ (IR−) mice (*n* = 9 and *n* = 13, respectively) at 12, 16, and (occasionally) 20 wk post-transplantation. However, none of these cytokines were present at detectable levels (limit of detection = <3.2 pg/ml).

Taken together, these results support the notion that the time-dependent appearance of the activated memory CD4^+^ T cell subset in hNOJ mice is due to HPE-type HSP.

### Expression Profiles of CCR5 and CXCR4 in CD4^+^ T Cells in hNOJ Mice

**Figure 6:**
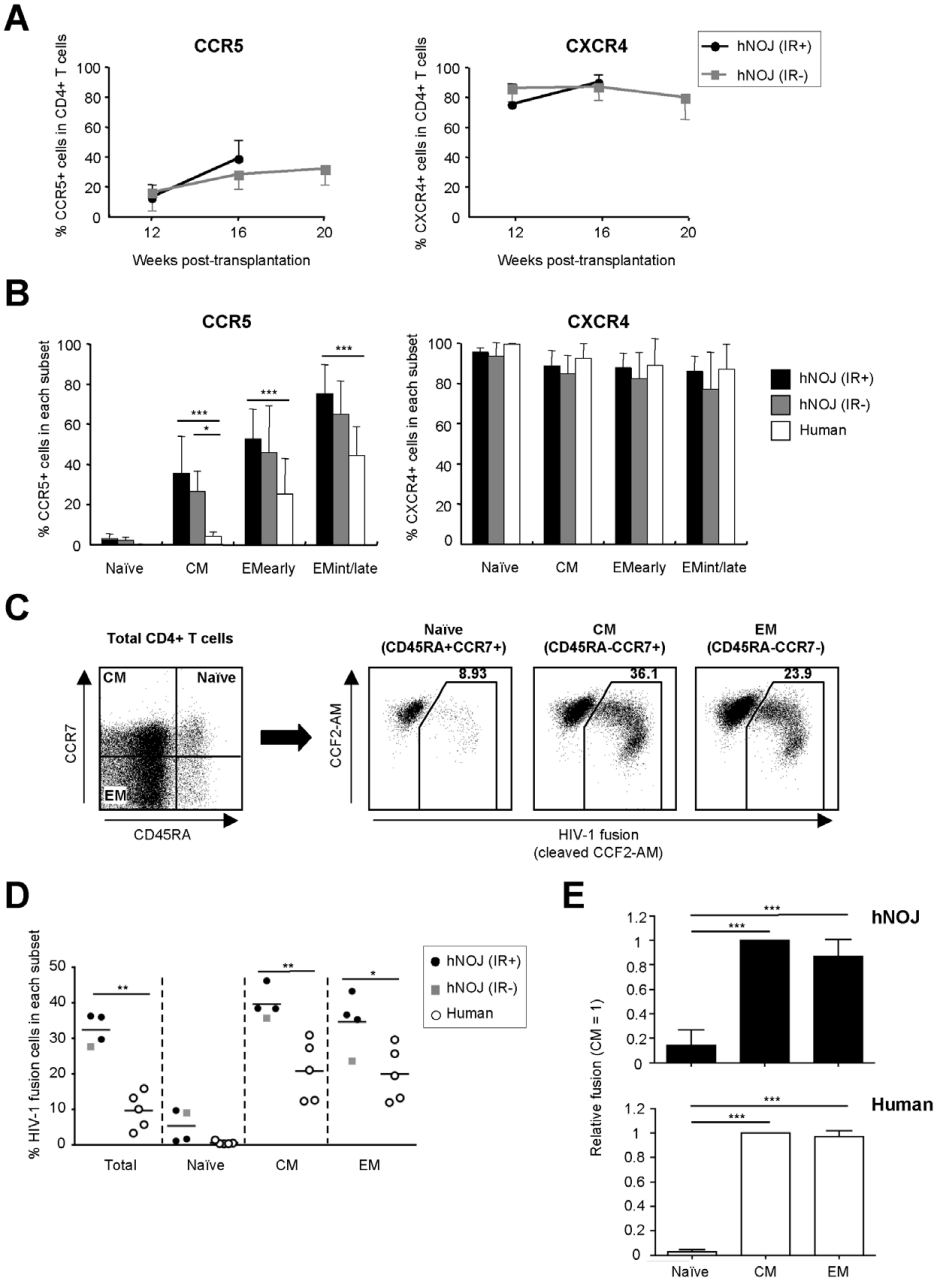
HIV-1 co-receptors, CCR5 and CXCR4, expression profiles and *ex vivo* R5 HIV-1 infectivity of CD4^+^ T Cells in hNOJ mice. (A) Changes in the percentage of CCR5^+^ (left) and CXCR4^+^ (right) cells within the peripheral blood CD4^+^ T cell population isolated from hNOJ (IR+) and hNOJ (IR−) mice (*n* = 18 and *n* = 6, respectively). Data are expressed as the mean ± SD. (B) Percentage of CCR5^+^ and CXCR4^+^ cells within the naïve, CM, EM_early_, and EM_int/late_ subsets of peripheral blood CD4^+^ T cells isolated from hNOJ mice at 16 wk post-transplantation (*n* = 18 and *n* = 6, respectively) and from humans (*n* = 5). Data are expressed as the mean ± SD. Significant differences (^*^ *P*<0.05, ^***^ *P*<0.001) were determined by two-way factorial ANOVA with the Bonferroni multiple comparison test. (C, D, E) Fusion assay using R5 HIV-1 and CD4^+^ T cells. Splenic CD4^+^ T cells from hNOJ mice at ≥17 wk post-transplantation (*n* = 4; three hNOJ (IR+) mice and one hNOJ (IR−) mouse) or peripheral blood CD4^+^ T cells from humans (*n* = 5) were infected *ex vivo* with HIV-1_NL-AD8-D-BlaM-Vpr_. (C) Naïve, CM, and EM subsets of CD4^+^ T cells (gated on CD3^+^CD4^+^CD8^−^) were defined as CD45RA^+^CCR7^+^, CD45RA^−^CCR7^+^, and CD45RA^−^CCR7^−^, respectively, by flow cytometry. (D) Percentage of R5 HIV-1 fusion cells within the total CD4^+^ T cell population and within the naïve, CM, and EM subsets in hNOJ mice and humans. Individual data points are plotted. The black lines represent the mean. Significant differences (^*^ *P*<0.05, ^**^ *P*<0.01) were determined by the unpaired *t* test. (E) Relative ratio of R5 HIV-1 fusion among the naïve, CM, and EM subsets from hNOJ mice and humans. The level of R5 HIV-1 fusion in each of the CD4^+^ T cell subsets relative to that in the corresponding CM subset. Data are expressed as the mean ± SD. Significant differences (^***^ *P*<0.001) were determined by Tukey’s multiple comparison test.

The expression profiles of the HIV-1 co-receptors, CCR5 and CXCR4, on CD4^+^ T cells within the PBMC populations in hNOJ mice and humans were compared by flow cytometry. The percentage of CCR5^+^CD4^+^ T cells gradually increased over time in both hNOJ (IR+) and hNOJ (IR−) mice (*n* = 18 and *n* = 6, respectively) (Figure 6A, left panel). Consistent with this, a substantial percentage of CCR5^+^ cells was observed in both hNOJ (IR+) and hNOJ (IR−) mice (*n* = 18 and *n* = 6, respectively) and humans (*n* = 5), depending on the differentiation status of the CD4^+^ T cells (Figure 6B, left panel). However, clear differences between hNOJ mice and humans were observed. For example, more CCR5^+^ cells were present within the CD4^+^ T cell population in hNOJ mice than in humans, and the CM subset in hNOJ mice comprised 30−40% CCR5^+^ cells, whereas in humans it comprised significantly fewer CCR5^+^ cells (Figure 6B, left panel). In contrast to CCR5, CXCR4 expression was consistently observed on approximately 80% of CD4^+^ T cells in both hNOJ (IR+) and hNOJ (IR−) mice (*n* = 18 and *n* = 6, respectively) throughout the course of the experiment (Figure 6A, right panel), although the percentage of CXCR4-expressing cells at an advanced differentiation stage tended to be slightly lower in both hNOJ (IR+) and hNOJ (IR−) mice (*n* = 18 and *n* = 6, respectively) (Figure 6B, right panel).

### *Ex vivo* R5 HIV-1 Infectivity of Reconstituted CD4^+^ T Cells

We next performed *ex vivo* experiments based on fusion assays using reconstituted CD4^+^ T cells to examine the susceptibility to R5 HIV-1 infection. CD4^+^ T cells were prepared from the spleens of hNOJ mice during the later phase (≥17 wk) post-transplantation (*n* = 4; one hNOJ (IR+) and three hNOJ (IR−) mice) or from human PBMCs (*n* = 5), and infected with R5 HIV-1 containing a Vpr/β-lactamase fusion protein (HIV-1_NL-AD8-D-BlaM-Vpr_). Because of the limited number of detector channels on the flow cytometer, each CD4^+^ T cell subset was defined as naïve (CD45RA^+^CCR7^+^), CM (CD45RA^−^CCR7^+^), or EM (CD45RA^−^CCR7^−^) in this experiment, and R5 HIV-1 fusion cells were detected within each subset (Figure 6C). In parallel with the low percentage of CCR5 expression, R5 HIV-1 fusion was rarely observed in the naïve subsets from both hNOJ mice and humans, confirming the CCR5-dependent infection of reconstituted CD4^+^ T cells by R5 HIV-1.

As shown in Figure 6D, the CM and EM subsets from hNOJ mice contained significantly more R5 HIV-1 fusion cells than those derived from humans. When the amount of R5 HIV-1 fusion within each CD4^+^ T cell subset was expressed as a value relative to that of the corresponding CM subset (which was set at 1), we found that the CM and EM subsets in both hNOJ mice and humans were highly susceptible to R5 HIV-1 compared with the naïve subset, indicating that the susceptibility to R5 HIV-1 among the CD4^+^ T cell subsets was similar in hNOJ mice and humans (Figure 6E). Notably, despite the finding that the percentage of CCR5-expressing cells in the CM subset was nearly half that in the EM subsets (Figure 6B, left panel), R5 HIV-1 fused efficiently to both the CM and EM subsets (Figure 6D and 6E). This result is not surprising, as similar observations were also made in a simian immunodeficiency virus infection model [43]. Furthermore, the CCR5^−^ CM subset expresses CCR5 mRNA and is very susceptible to R5 virus [44].

### *In vivo* R5 HIV-1 Infection in hNOJ Mice

To investigate whether the different constitution of CD4^+^ T cell subsets, i.e., naïve- or memory subset-rich, affected HIV-1 infectivity *in vivo*, we used hNOJ (IR+) mice at 10 wk post-transplantation (*n* = 7) as the naïve-rich group and hNOJ (IR−) mice at ≥12 wk post-transplantation (*n* = 8) as the memory-rich group. After the hNOJ mice were challenged intravenously with R5 HIV-1 (HIV-1_NL-AD8-D_), the plasma viral load was monitored weekly by quantitative real-time RT-PCR up until 5 wk post-challenge.

**Figure 7:**
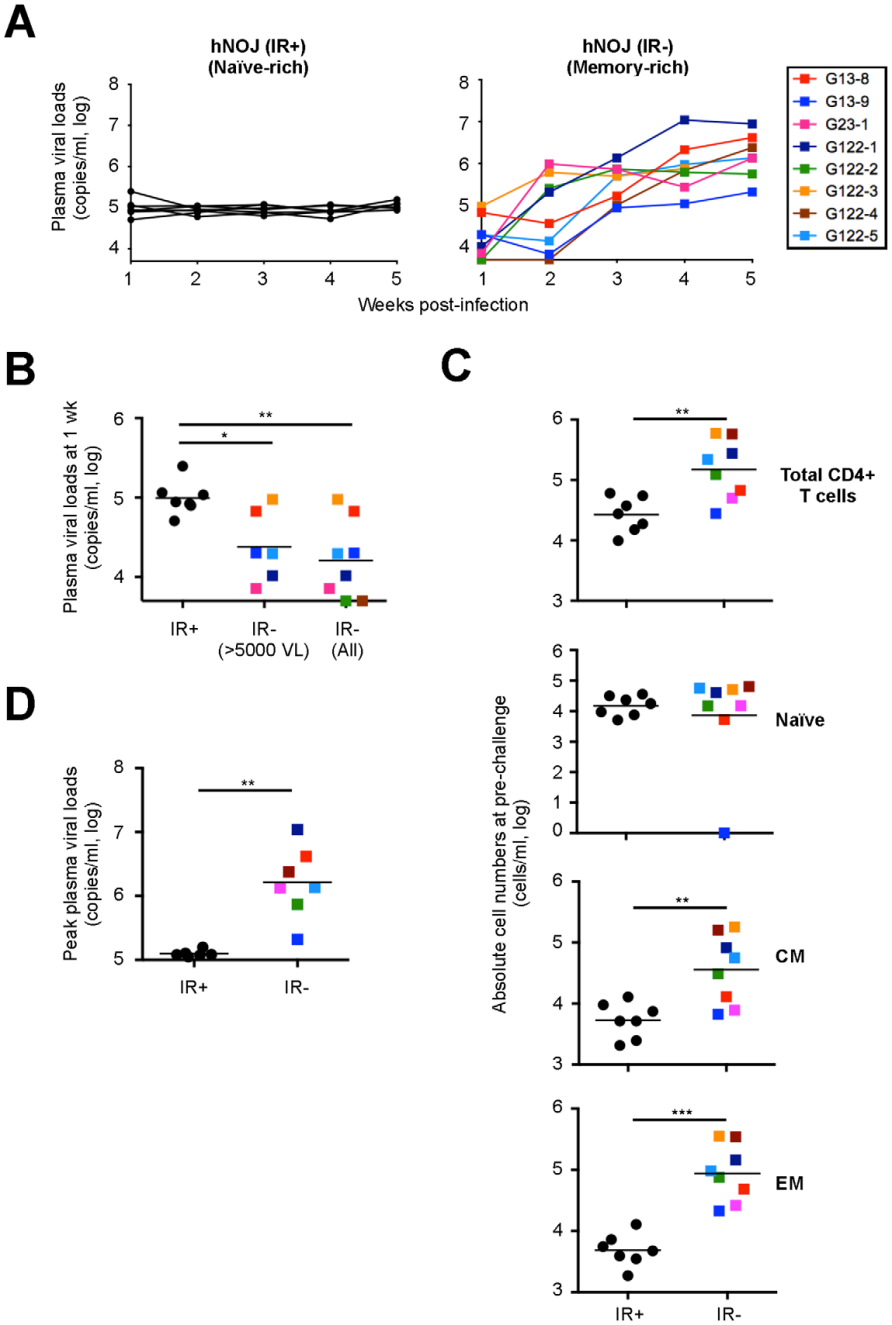
*In vivo* R5 HIV-1 infection in hNOJ mice. hNOJ mice were challenged intravenously with HIV-1_NL-AD8-D_ and divided into two groups: naïve-rich hNOJ (IR+) mice at 10 wk post-transplantation (*n* = 7) and memory-rich hNOJ (IR−) mice at ≥12 wk post-transplantation (*n* = 8), based on the percentage of each individual CD4^+^ T cell subsets at pre-challenge. (A) Weekly analysis of the plasma viral load. Individual hNOJ (IR−) mice are denoted by different colors in this and in the following figures. (B) The plasma viral load at 1 wk post-challenge. Data are plotted individually along with the mean (black lines). Significant differences (^*^ *P*<0.05, ^**^ *P*<0.01) between hNOJ (IR+) mice (*n* = 7) and hNOJ (IR−) mice in which the plasma viral load was detectable (>5000 VL, *n* = 6) or all hNOJ (IR−) mice (*n* = 8) were determined by the Mann-Whitney U test. (C) The absolute number of CD4^+^ T cells in the peripheral blood at pre-challenge [hNOJ (IR+) mice; *n* = 7 and hNOJ (IR−) mice; *n* = 8]. Each CD4^+^ T cell subset (Naïve, CM, and EM) was defined as outlined in the legend to Figure 6. Data are plotted individually along with the mean (black lines). Significant differences (^**^ *P*<0.01, ^***^ *P*<0.001) were determined by the Mann-Whitney U test. (D) The peak plasma viral load during 5 wk post-challenge [hNOJ (IR+) mice; *n* = 6 and hNOJ (IR−) mice; *n* = 7]. Data are plotted individually along with the mean (black lines). Significant differences (^**^ *P*<0.01) were determined by the Mann-Whitney U test.

Viral RNA was detected in the plasma of all naïve-rich hNOJ (IR+) mice at 1 wk post-challenge, and was maintained at around 1×10^5^ copies/ml over time (Figure 7A, left panel). On the other hand, memory-rich hNOJ (IR−) mice showed a blunted plasma viral load during the early phase post-challenge; however, the viral load reached peak values that were approximately 1-log higher than those in naïve-rich hNOJ (IR+) mice by 5 wk post-challenge (Figure 7A, right panel). Of note, the viral load was undetectable in the plasma of two (G122-2 and G122-5) of the eight memory-rich hNOJ (IR−) mice by 1 and 2 wk post-challenge, respectively, and the mean value for the plasma viral load at 1 wk post-challenge in memory-rich hNOJ (IR−) mice was significantly lower than that in naïve-rich hNOJ (IR+) mice (Figure 7B). Surprisingly, when we compared the absolute number of peripheral blood CD4^+^ T cells between naïve-rich hNOJ (IR+) and memory-rich hNOJ (IR−) mice just before challenge, we found that memory-rich hNOJ (IR−) mice had significantly more CD4^+^ T cells (apart from for the naïve subset) than naïve-rich hNOJ (IR+) mice (Figure 7C), indicating that the absolute number of peripheral blood CD4^+^ T cells at pre-challenge is not associated with the timing and level of the initial plasma viral load. However, the CD4^+^ T cell constitution in memory-rich hNOJ (IR−) mice may contribute to subsequent virus replication, because the peak value for the plasma viral load during 5 wk post-challenge was significantly higher in memory-rich hNOJ (IR−) mice than in naïve-rich hNOJ (IR+) mice (*n* = 6 and *n* = 7, respectively; one hNOJ (IR+) and one hNOJ (IR−) mice were excluded from this analysis as the experiment was interrupted before 5 wk post-challenge) (Figure 7D).

**Figure 8:**
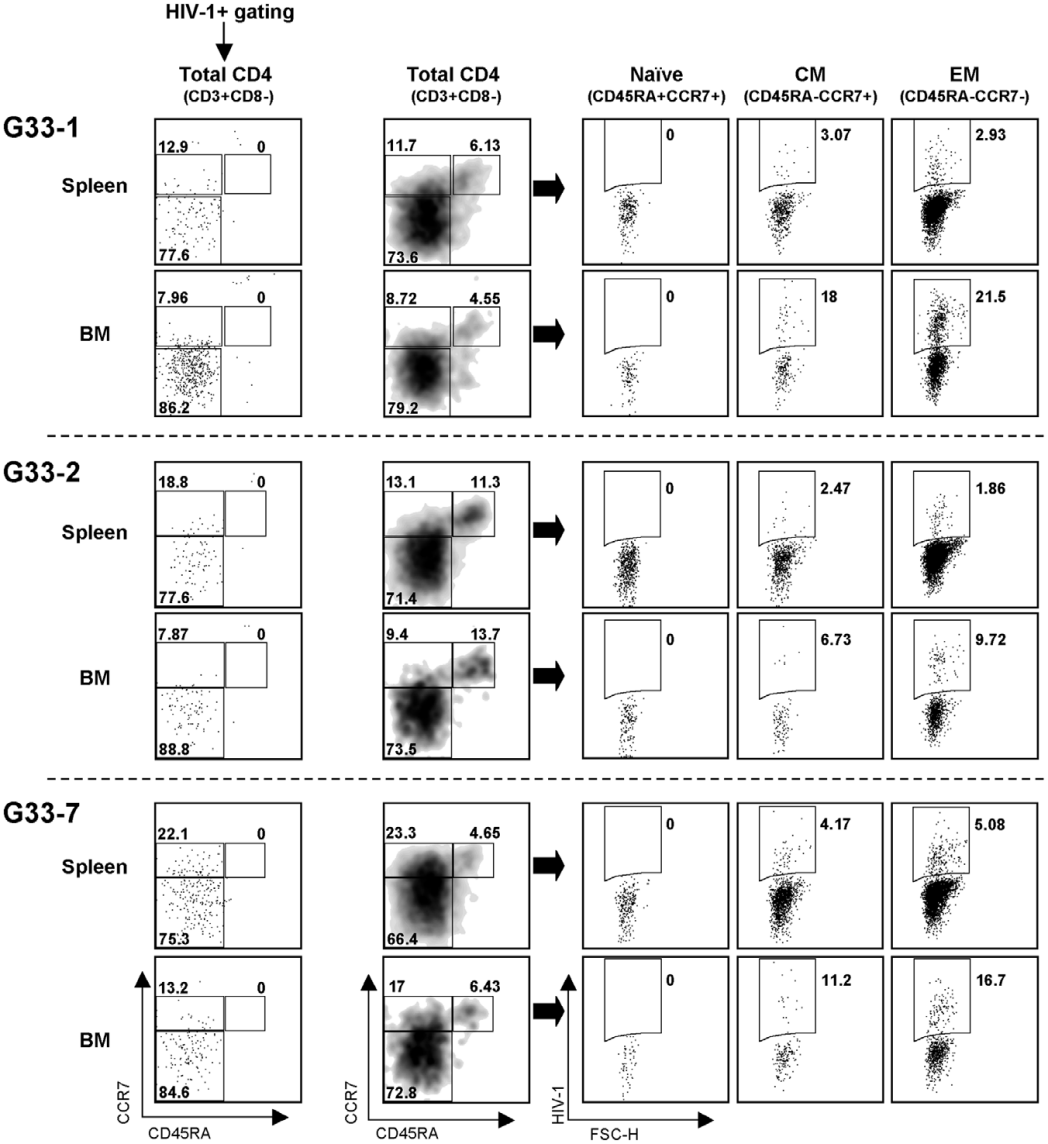
Identification of R5 HIV-1-infected cells in hNOJ mice. Three hNOJ (IR−) mice (G33-1, G33-2, and G33-7), all at 13 wk post-transplantation, were challenged intravenously with HIV-1_NL-AD8-D_. At 2 wk post-challenge, the mice were sacrificed and the infected cells in the spleens and BM were analyzed by flow cytometry. Each CD4^+^ T cell subset (Naïve, CM, or EM) was defined as outlined in the legend to Figure 6. Infected cells were identified based on their expression of the fluorescent reporter, DsRed.

To characterize the HIV-1-infected CD4^+^ T cells further, three additional hNOJ (IR−) mice were analyzed at 2 wk post-challenge with R5 HIV-1 (Figure 8). HIV-1 infected cells were detected according to their expression of the fluorescent reporter, DsRed, by flow cytometry as described previously [29], [45], and CD4^+^ T cells were rendered CD3^+^CD8^−^ cells in order to include CD4-downmodulated HIV-1-infected cells. When analyzing the spleens and BM, we detected infected cells within the CM and EM subsets, but not within the naïve subset. The majority of the infected cells were within the EM subset, mainly because the EM subset was the most numerous among the CD4^+^ T cells (CD3^+^CD8^−^ cells) in both the spleens and BM at 2 wk post-challenge. However, the percentage of infected cells within the CM and EM subsets was identical. These results indicate that both the CM and EM subsets are a target of R5 HIV-1 *in vivo*.

## Discussion

NOJ mice were recently developed as an alternative recipient mouse strain for the construction of humanized mice [18]. This novel strain was used in the present study. Since the Janus family tyrosine kinase, Jak3, mediates downstream signaling from the common γ chain, and is responsible for lymphocytes development [46], the phenotype of NOJ mice, which lack the *Jak3* gene, is the same as that of NOG/NSG mice [18]. Various methods are used to construct conventional humanized mouse models; however, the different methods may result in different outcomes in terms of hematopoietic cell development. Therefore, to make the experiment easier and more effective as previously suggested [9], we constructed humanized NOJ mice by transplanting CD34^+^ HSCs isolated from umbilical cord blood into the liver of newborn mice. The present study used hNOJ mice that were not irradiated prior to HSC transplantation (i.e., hNOJ (IR−) mice), in comparison with irradiation-treated hNOJ mice (i.e., hNOJ (IR+) mice) that have been already established [18], [47], [48]. Each hematopoietic cell population, such as T cells, B cells, monocytes, and dendritic cells in irradiation-treated hNOJ mice show the same developmental characteristics [18], [47], [48] as those in other conventional humanized mouse models constructed with different strains using the same method (newborn mice were irradiated prior to intrahepatic transplantation of umbilical cord blood-derived HSCs) [16], [17], [49]. Therefore, hNOJ mice may be an alternative humanized mouse model, and experimental data pertaining to these hNOJ mice may be shared with other conventional humanized mouse models. However, an obvious difference between NOJ mice and other strains is their susceptibility to irradiation, which means that they have a limited life-span.

To evaluate hNOJ mice as an experimental animal model of HIV-1 infection, we first investigated how the degree of reconstitution affects the cellularity and development of CD4^+^ T cells using hNOJ (IR+) and (IR−) mice, which represent high and low chimerism groups, respectively. However, we found that qualitative differences in the characteristics of the reconstituted CD4^+^ T cells, such as the process of differentiation, the degree of activation, and CCR5/CXCR4 expression, were similar between hNOJ (IR+) and hNOJ (IR−) mice at least during the first 16 wk post-transplantation. In hNOJ mice, the cellularity of CD4^+^ T cells and proportion of each CD4^+^ T cell subset within the peripheral blood were similar to those in other organs, such as the spleen and BM (Figure 4B and data not shown), suggesting that the peripheral blood CD4^+^ T cell population may be representative of the CD4^+^ T cell distribution throughout other systemic compartments in these mice. In humans, the CD45RA^+^CCR7^+^ naïve and CD45RA^−^CCR7^−^ EM subsets of CD4^+^ T cells constitute the major subset within the peripheral blood and spleen, respectively [50]. The most striking difference in the CD4^+^ T cells between hNOJ mice and humans noted here was that hNOJ mice showed a higher percentage of CD4^+^ T cells expressing HLA-DR, Ki-67, and CCR5 during the later phase post-transplantation.

The memory subset (either CD45RA-negative or CD45RO-positive) tends to increase with time in conventional humanized mice [28], [51], [52]. We found that the expansion of the CD45RA^−^CCR7^−^CD27^+^ EM_early_ and CD45RA^−^CCR7^−^CD27^−^ EM_int/late_ subsets, but not that of the CD45RA^−^CCR7^+^CD27^+^ CM subset, was associated with an activated phenotype. As suggested elsewhere [27], [28], this is probably due to the occurrence of HSP, particularly HPE-type HSP, because (1) memory subsets, particularly the EM_int/late_ subset, expressed the proliferation marker, Ki-67, at higher levels than the naïve subset, and the levels were much higher than those expressed by the EM_int/late_ subset within human PBMC population; (2) the EM_int/late_ subset showed the greatest IFN-γ-producing capacity; and (3) IL-2, IL-7, and IL-15 were undetectable in the plasma when CD4^+^ T cells converted to an activated memory phenotype.

HPE-type HSP requires TCR-dependent antigen recognition, which is independent of IL-7 [25], [26]. In line with this, Onoe *et al*. demonstrated that HPE-type HSP of CD4^+^ T cells correlated with the percentage of CD14^+^ monocytes in the periphery when autologous T cells reconstituted in BLT mice were adoptively transferred into T cell-deficient humanized NOD/SCID mice and suggested that CD4^+^ T cells need to interact with self MHC-II molecules expressed by autologous myeloid-derived antigen-presenting cells (APCs) to drive their expansion [26]. Furthermore, Suzuki *et al*. used a humanized mouse model based upon HLA-DR-transgenic NOG sub-strain mice to show that expansion of the EM subset of CD4^+^ T cells occurs in an MHC-II (HLA-DR)-dependent manner [28]. In the present study, hNOJ (IR+) mice showed a significantly higher proportion of CD14^+^ monocytes within the PBMC population, and developed more CD4^+^ T cells than hNOJ (IR−) mice. Although we did not examine the proportion of APCs in hNOJ (IR+) or hNOJ (IR−) mice in the present study, it is conceivable that hNOJ (IR+) mice have more APCs due to the higher level of chimerism. Therefore, TCR-dependent antigen recognition via myeloid-derived APCs may enhance HPE-type HSP of reconstituted CD4^+^ T cells in hNOJ (IR+) mice.

The main aim of this study was to examine whether, and how, HIV-1 infectivity is affected in different humanized mouse models in which the CD4^+^ T cell composition is qualitatively and/or quantitatively different, e.g., naïve- or memory-rich phenotypes. The results showed that the infectivity of R5 HIV-1 was different between the naïve-rich and memory-rich mouse groups. One of the most noticeable differences between the two mouse groups was that the plasma viral load at 1 wk post-challenge was significantly higher in naïve-rich hNOJ (IR+) mice than it was in memory-rich hNOJ (IR−) mice. Although robust plasma viral loads have been reported in other conventional humanized mouse models of R5 HIV-1 infection even at 1 wk post-challenge [17], [53], [54], our result may be curious because the R5 HIV-1 infectivity of naïve CD4^+^ T cells is very low, and because naïve-rich hNOJ (IR+) mice would have few memory CD4^+^ T cells compared with memory-rich hNOJ (IR−) mice. In the present study, we could not determine which cellular parameters pertaining to CD4^+^ T cells at pre-challenge were associated with the initial plasma viral load; therefore, the reason for the difference in viral load remains unclear. One possibility may be the different proportions of dendritic cells present in hNOJ (IR+) and hNOJ (IR−) mice, since HIV-1 transmission/dissemination efficiently occurs via dendritic cell-CD4^+^ T cell contact [3], [55], [56], [57]. This needs to be clarified in future studies.

Another noticeable difference between naïve-rich hNOJ (IR+) and memory-rich hNOJ (IR−) mice in terms of the viral infection was the peak level of the plasma viral load. Memory-rich hNOJ (IR−) mice showed a markedly higher peak level of the plasma viral load during 5 wk post-challenge, suggesting the contribution of the memory CD4^+^ T cell subset in this setting. Notably, Nie *et al*. showed massive infection of the CD45RA^−^CD45RO^+^CCR7^−^ EM subset by R5 HIV-1 in NOG-background humanized mice [51]. Likewise, when we analyzed the spleens and BM from hNOJ (IR−) mice at 2 wk post-challenge with R5 HIV-1, the majority of infected cells were present in the EM subset, most likely because the EM subset was the most numerous at that time. However, when individual subsets were analyzed carefully, the percentage of infected cells in the CM and EM subsets was similar. This is explained by the fact that the fusion efficiency of R5 HIV-1 was comparable between the two subsets (Figure 6E). Taken together, the data obtained in the present study suggest that the CM subset plays a role as both a target and a reservoir for R5 HIV-1 in an HIV-1 infected humanized NOJ mouse model.

hNOJ (IR+) and hNOJ (IR−) mice were highly permissive for infection with both CCR5-tropic HIV-1 (Figures 7 and 8) and CXCR4-tropic HIV-1 (data not shown). However, because hNOJ (IR+) mice have such a short life-span due to irradiation, their use is restricted to the early phase post-transplantation; their use, therefore, is restricted to the study of short-term of HIV-1 infection. By contrast, hNOJ (IR−) mice would be useful for longitudinal analyses of HIV-1 infection, as shown by other studies that used other conventional humanized mouse models [22], [53], [58]. However, it should be noted that level of HIV-1 infectivity would change, depending on the cellularity of the reconstituted CD4^+^ T cells in hNOJ mice. Therefore, the selective use of humanized mice according to the disease model is important. For instance, hNOJ mice at the early phase post-transplantation would be a useful model for healthy young humans, in which the naïve CD4^+^ T cell subset is relatively rich [59], [60], [61]. On the other hand, hNOJ mice at the late phase post-transplantation (i.e., hNOJ (IR−) mice), might be more suitable as a model for Immune Reconstitution Inflammatory Syndrome, which involves an activated CD4^+^ T cell burst during post anti-retro viral therapy in HIV-infected individuals [62].

In conclusion, although no obvious differences in the cellularity of CD4^+^ T cells were found between hNOJ (IR+) and hNOJ (IR−) mice, at least within 16 wk post-transplantation, reconstituted CD4^+^ T cells converted to an activated memory phenotype over time. The infectivity of R5 HIV-1 was modulated *in vivo* in these humanized mice, depending on the percentage of memory CD4^+^ T cells. Therefore, the present study suggests that humanized mouse models should be used selectively according to the experimental objectives, taking into account different human physiological states, to gain an appropriate understanding of HIV-1 infection/pathogenesis.
